# A Primer on Integral Theory and Its Application to Mental Health Care

**DOI:** 10.1177/2164956120952733

**Published:** 2020-09-21

**Authors:** James D Duffy

**Affiliations:** 1 Department of Clinical Psychiatry, University of California at San Francisco, San Francisco, California

**Keywords:** integral, mental health care, paradigm, wisdom

## Abstract

Contemporary psychiatry has become increasingly focused on biological treatments. Many critics claim that the current paradigm of psychiatry has failed to address the escalating mental health-care needs of our communities and may even be contributing to psychopathology and the burden of mental illness. This article describes the foundations of Integral Theory and proposes that this model offers a framework for developing integral psychiatry and a more effective and compassionate mental health-care system. An integral model of psychiatry extends biopsychosocial approaches and provides the scaffolding for more effective approaches to integrative mental health care. Furthermore, rather than focusing on psychopathology, the Integral theory model describes the emergence of human consciousness and supports a mental health-care system that addresses mental illness but also promotes human flourishing.

## Introduction

There has never been a period in human history when so many diverse perspectives have demanded expression on the local and world stage. Fuelled by rapidly evolving information technologies, and emboldened by their access to powerful cataclysmic weapons, multiple ethnic populations and demographic groups are demanding to be heard. The complexity of these competing worldviews can be confusing, even overwhelming at times. This escalating complexity is not limited to political systems and is manifesting in all areas of human endeavor including health care.

In concert with these diverse perspectives, medical science is unleashing staggering new treatments that raise multiple ethical challenges. Patients are excited about these scientific miracles but also appropriately concerned that their personal beliefs and preferences will be respected. The Integral model provides a framework for understanding how we can navigate these myriad perspectives and potentials, effectively and respectfully.

## Integral Theory

Integral theory, as described by the contemporary American philosopher Ken Wilber, is essentially a philosophical map that brings together more than 100 ancient and contemporary theories in philosophy, psychology, contemplative traditions, and sociology. Rather than attempting to describe “the one correct view,” Integral theory attempts to describe a framework for understanding and valuing the perspective of each theory and philosophical tradition and understanding how they relate to one another. Through this respectful and integrating worldview, Integral theory recognizes the evolutionary impulse that incorporates, rather than devalues or destroys, previous perspectives. The integral worldview therefore includes the essential perspectives of prerational, traditional, modernist, and postmodernist worldviews but also recognizes the limitations of each of these worldviews in addressing the increasingly complex challenges manifesting in the 21st century. Integral theory extends upon postmodernism by moving beyond its core construct of deconstructionism (and the absence of an absolute truth) to a constructivist viewpoint that recognizes that all worldviews have validity in the context of the evolutionary stage and local conditions within which they are manifesting. This constructionist approach therefore enables one to understand and work skillfully with all the worldviews that are simultaneously manifesting in an interconnected 21st century world—whether this is in a nongovernmental agency or the clinician’s office.

The term, “Integral” has been used by several philosophers over the past 2 centuries. However, Ken Wilber has been the most influential proponent of this term and has expanded the philosophical foundations. Through his review of all major philosophic and religious traditions, Wilber writes:

Integral theory describes a comprehensive map that pulls together multiples includes comprehensive, inclusive, non-marginalizing, embracing. Integral approaches to any field attempt to be exactly that: to include as many perspectives, styles, and methodologies as possible within a coherent view of the topic. In a certain sense, integral approaches are “meta-paradigms,” or ways to draw together an already existing number of separate paradigms into an interrelated network of approaches that are mutually enriching.^1^

I don’t believe that any human mind is capable of 100 percent error. So instead of asking which approach is right and which is wrong, we assume each approach is true but partial, and then try to figure out how to fit these partial truths together, how to integrate them—not how to pick one and get rid of the others.^2^

### Why Explore a New Framework for Mental Health Care?

Psychiatry faces considerable challenges that are not being adequately addressed by our current models of mental health care. It can be reasonably argued that we are experiencing a major crisis in mental health that may threaten our ability to maintain stable societal systems. These challenges include the following:

1. The rapid increase in mental illness. The suicide rate in the United States has increased 31% during the period from 2001 to 2017 from 10.7 to 14.0 per 100,000 and we are witnessing increasing levels of mental health disorders in our populations.^3,4^ Although there are several sociocultural factors influencing this trend, these data indicate that the current biological allopathic psychiatry paradigm has proven itself to be inadequate to addressing this escalating challenge.
2. Biological psychiatry is exploring the clinical utility of potent new therapies (eg, entheogens) that hold the potential for dramatic effects on human consciousness, both positive and negative.
3. The population is becoming increasingly tethered to information interfaces (eg, smartphones) that have been shown to produce behavioral changes and physical changes in neural structures underlying social and metacognitive functions.^5^
4. Despite the rise of so-called “social media,” the data indicate that individuals are experiencing increased loneliness, with its accompanying negative impact on mental health.^6^
5. Complementary (ie, nonallopathic) approaches are gaining increased acceptance among the community.^7^
6. Health-care professionals are experiencing escalating levels of burnout that is not understood or effectively treated by the current mental health-care model.^8^
7. Communities are not prepared to meet the societal upheavals that are inevitable with the emerging dominance of artificial intelligence technologies.
8. Recent advances in genetics will provide scientists with the ability to potentially radically reshape the human genome and current models of bioethics are simply inadequate to contain this emerging “god-like” capacity.

## Limitations of the Biopsychosocial Model

It is reasonable to question whether the Integral model simply represents a repackaging of the biopsychosocial model (BPS) first proposed by George Engel in 1977 as alternative to reductionist biomedical models. The BPS has certainly gained widespread acceptance and has been helpful in supporting more eclectic and holistic approaches to understanding the pathogenesis and treatment of mental illness. There are, however, several limitations to the model, specifically the BPS model as follows:

1. Describes domains of function and intervention rather than perspective or etiology. This makes the model vulnerable to being shaped by the dominant biological reductionism that attempts to describe all domains in objective metrics, for example, social neuroscience rather sociology.
2. Does not provide any insights into how each domain relates to one another.
3. Does not provide any common language for different professionals to communicate effectively across disciplines.
4. Does not provide any descriptions of the different stages, states, and lines of human experience.
5. Rather than replacing the BPS model, Integral theory (as described later) extends and deepens the BPS model to include a deeper appreciation of the importance of promoting human flourishing and not simply combating human pathology.

## Limitations of Integrative Medicine Model

“The limits of my language mean the limits of my world”*—*Ludwig Wittgenstein (1889–1951).

It is important to distinguish between the Integral theory model and “integrative medicine.” Although there has been an increasing interest in the so-called “integrative” approaches to health care, the definition of integrative medicine remains unclear. The Academic Consortium of Academic Medical Centers in Integrative medicine states:

Integrative medicine and health reaffirms the importance of the relationship between practitioner and patient, focuses on the whole person, is informed by evidence, and makes use of all appropriate therapeutic and lifestyle approaches, healthcare professionals and disciplines to achieve optimal health and healing. ^9^

This definition is somewhat helpful in describing the operational characteristics of integrative medicine and does have some heuristic value. However, it fails to address the key challenge facing any “integrative team,” that is, “what is your perspective, what is my perspective, and do they relate to one another.” Unfortunately, this inability to define and respect various perspectives has meant that most integrative health-care systems are driven by the idiosyncrasies of a particular clinician, typically an allopath.

## The Origins of Integral Theory

The intellectual lineage of contemporary Integral Theory includes philosophers, psychologists, and sociologists dating back more than 2 centuries.

### Georg Hegel (1770–1831)

Georg Hegel can justifiably considered the first “integral philosopher.” Contrary to Kant, Hegel described knowledge and consciousness as creating a persistent dynamic dialectic tension that impels consciousness to evolve across distinct stages. He suggested that the evolution of human consciousness mirrored the larger impulse of the universe to move toward the absolute. Hegel suggested that each evolutionary stage incorporated, and did not destroy, the previous stages. He wrote, “every era’s world view was both a valid truth unto itself and also an imperfect stage in the larger process of absolute truth’s unfolding.”^10^ Through his description of an inclusive model of evolutionary consciousness, Hegel can rightfully considered the first “Integral Philosopher.”

### Sri Aurobindo (1872–1950)

The term *integral* was first used in the context of psychology in 1914 by the Indian sage Sri Aurobindo when he described integral yoga as the process of the uniting of all the parts of one’s being with the Divine, and the transformation of all the developmental states of consciousness, emotions, intellect, and physical states into ultimate harmony.^11^ Indra Shen (1903–1994) reframed Aurobindo’s ideas into an “Integral Psychology” model that he proposed in contrast to the reductionist behavioral and psychoanalytic paradigms that dominated Western psychology at that time. ^12^

### Jean Gebser (1905–1973)

The Swiss phenomenologist and interdisciplinary scholar Jean Gebser independently introduced the term integral to describe his model of the evolution of human consciousness. In his influential book, *The Ever-Present Origin,* ^13^ Gebser described history as the punctuated evolution of human consciousness along 5 distinct structures of consciousness such as archaic, magic, mythical, mental, and integral.

### James Mark Baldwin (1861–1934)

Baldwin was one of the first psychologists to study the intellectual and emotional development of children. He refined the constructs of human development by describing the dialectic development of human consciousness along distinct stages, that is, the prelogical, logical, extra-logical, and hyper-logical stages. Other developmental psychologists including Piaget, Kohlberg, Loevinger, Gilligan, Gardner, and Kegan expanded Baldwin’s insights.^14^

### Abraham Maslow (1908–1970)

Abraham Maslow exerted a powerful influence in several areas of psychology. He described a hierarchy of humans beginning with survival and culminating in self-actualization. Maslow coined the term “positive psychology” and highlighted the importance of recognizing and supporting each person’s drive toward their innate potential. In this way, he was an intellectual progenitor to Integral theory. This focus is captured in his statement: “It is as if Freud supplied us the sick half of psychology and we must now fill it out with the healthy half.”^15^

### Clare Graves (1914–1986)

Clare W Graves was a professor of psychology at Union College in Schenectady, New York. He developed an epistemology of human psychology based on his study of undergraduate students at the university. Graves described a hierarchy of human development that described the emergence of human consciousness across specific stages.

The psychology of the adult human being is an unfolding, ever-emergent process marked by subordination of older behavior systems to new, higher order systems. The mature person tends to change his psychology continuously as the conditions of his existence change. Each successive stage or level of existence is a state through which people may pass on the way to other states of equilibrium. When a person is centralized in one of the states of equilibrium, he has a psychology, which is particular to that state. His emotions, ethics and values, biochemistry, state of neurological activation, learning systems, preference for education, management, and psychotherapy are all appropriate to that state. 16

### Ken Wilber (1949–)

Ken Wilber is an independent philosopher who has surveyed and integrated many of the world’s philosophic and religious traditions to develop a comprehensive Integral model. Integral theory is a meta-theory that attempts to integrate all human wisdom into a new, emergent worldview that is able to accommodate the perspectives of all previous worldviews, including those that may appear to be in contradiction to one another. The Integral model continues to expand in complexity and has been applied to many areas such as business, politics, ethics, religion, psychology, and philosophy. Wilber states:

I therefore sought to outline a philosophy of universal integralism. Put differently, I sought a world philosophy—an integral philosophy—that would believably weave together the many pluralistic contexts of science, morals, aesthetics, Eastern as well as Western philosophy, and the world’s great wisdom traditions. Not on the level of details—that is finitely impossible; but on the level of orienting generalizations: a way to suggest that the world really is one, undivided, whole, and related to itself in every way: a holistic philosophy for a holistic Kosmos, a plausible Theory of Everything.^17^

## A Brief Overview Wilber’s Integral Model

This section provides a brief overview of the Integral model. Ken Wilber has described an integral model that includes 5 elements that describe the organizing patterns of all reality.

Wilber’s Integral model is often referred to as the “AQAL” model that stands for all quadrants, all levels, all lines, all states, and all types. These 5 elements represent all the aspects through which we can describe individual and group manifestations and experiences. This 5 element framework organizes all potential ways of understanding and responding to any particular life circumstance and therefore enables one to select the most relevant and effective strategies for responding to that life circumstance.^1^

Here is brief description of each the 5 elements of the AQAL model.

## All Quadrants: The Basic Dimension Perspectives

**Figure 1.**
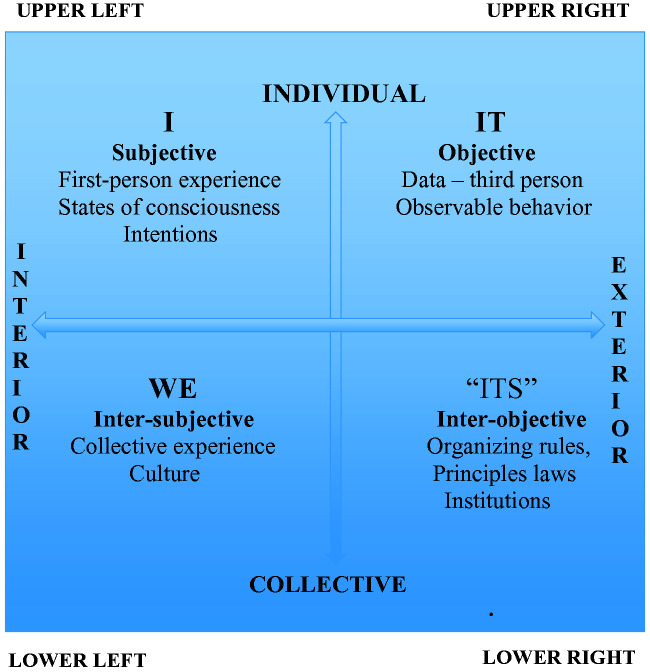
The Four Perspective Quadrants Described in Integral Theory.

Integral theory describes that all life conditions are filtered through 4 irreducible perspectives that come from one of “inside versus outside” (ie, subjective, intersubjective, objective, and interobjective perspectives) and “singular versus plural” perspectives. This describes 4 quadrants from which to perceive any life circumstance at any particular moment. You cannot understand one of these realities through the lens of any of the others and all 4 perspectives offer a partial and complementary perspective (rather than contradictory perspectives). It is interesting to note that these perspectives are included in almost all most languages, suggesting that they have universal applicability to human experience. According to Wilber, the 4 quadrants are as follows:

- *The “I” perspective*—The upper left quadrant (LUQ). This represents the individual’s first-person subjective experience (characterized as aesthetics and experiential consciousness). This quadrant contains all first-person experience of the inner stream of consciousness from bodily sensations, thoughts, soul, and spirit.
- *The “We” perspective*—The lower left quadrant (LLQ). This represents the social perspective—the inside of the collective intersubjective realm (characterized by shared values and cultural perspectives).
- *The “It” perspective*—The right upper quadrant (RUQ). This represents the third-person perspective (characterized by scientific objective third-person data).
- *The “Its” perspective*—The right lower quadrant (RLQ). This represents external (ecological) structures (characterized by social, regulatory, and political systems; Figure 1).

Wilber suggests that modern western society (and Western allopathic medicine) has become blinkered on the RUQ (the exterior objective perspective). This perspective only values facts that can be generated through the scientific method and marginalizes, devalues, or even denies the validity of first-person experience. This blinkered perspective clearly has very significant implications for psychiatry and psychology that attempt to understand the human psyche. Fortunately, the recent emergence of contemplative neuroscience (as the application of scientific method to studying the first-person phenomenology of contemplative practices) represents a major step toward linking interior and exterior domains.

## Levels

The levels of development represent stages of organization (or complexity) within a quadrant. The levels in each quadrant demonstrate part (a Holon) of the whole (holarchy), much like a “Russian doll” with each new level transcending the limitations of the previous levels while still including the essential aspects of each prior level.

Rather than replacing previous levels, each emergent level expands the complexity and capacity. This describes the emergence of “holons within a holarchy,” each one distinct but still part of a whole. This suggests that systems evolve in a punctuated way , for example, atoms to molecules to organisms.

Integral theory describes between 8 and 10 levels, depending upon the quadrant being described. The anthropologist, Jean Gebser, described 5 levels (ie, archaic, magic, mythic, rational, and integral) while Robert Kegan, Clare Graves, Jane Loevinger, and Erik Erikson have proposed other models. Each of these has validity depending upon which line they are describing and within which particular domain.

Spiral Dynamics (SD) theory of levels has found increasing recognition as a practical model for understanding the perspectives experienced at different levels of development. SD has grown out of the initial work by Clare W Graves that has been elaborated by Don Beck.^18^ The amalgam of Integral theory and SD theories is referred to Spiral Dynamics Integral (SDI). This model expands our understanding of the “values line” and how this can be understood in individuals and communities. In SD, the term “meme” refers to these core value systems. SDI describes *how* people think, and not *what* they think about. Our values describe the lens through which individuals or groups experience what is important, and therefore what motivates their actions. These value systems are shaped by the local conditions and individuals and groups can manifest different values (and responses) under different circumstances (eg, when faced with a situation that challenges their survival vs a situation that is less threatening). Any group is likely to manifest the value system of its majority. However, individuals may still possess their own values within the larger group—albeit typically under pressure to conform to the group values.

## Levels of Values Development Described by the SD Model

SD describes 6 “first-tier” levels (describing survival or reactive levels of being) and the 2 “second tier” value levels (describing flourishing or reflective levels of being). In an attempt to avoid any hierarchical implications, and to facilitate communication, specific colors have been assigned to each of the levels.

### First-Tier Value Levels

1. *The Archaic-Instinctual Level (Beige):* The primary values at this level are organized around basic survival such as food, sex, and housing.

 Manifested in the following: earliest hunter–gatherer groups, newborn infants, patients with advanced dementia, individuals experiencing severe deprivation, and social disconnection (eg, some people with serious mental illness who are living on the streets).

2. *Magical-Animistic (Purple):* Values are organized around magical spirits and thinking. The “spirits” exist in ancestors who bond the group together.

Manifested in the following: tribal groups, gangs, some corporate “tribes,” and individuals experiencing psychosis.

3. *Power Oriented (Red):* Values are organized around a drive to manifest personal authority in a world perceived as threatening and where there can be only one winner. The person at this stage seeks dominance and the total submission of others to their will. They do not experience remorse or concern for others perceived to be weaker than them.

Manifested in the following: dictators, gang leaders, malignant sociopaths, children at the “terrible twos” stage.

4. *Mythic Order (Blue)*: Values organized around belief in a benign and all-powerful higher authority that requires their rigid adherence to dualistic morality. This is often manifested in monotheistic religious structures that prescribe strict rules of conduct and subservience to an anointed hierarchical system.

Manifested in the following: Monotheistic religious fundamentalism, totalitarian societies, organizations, or societies with strict codes of ethics (such as certain professional groups and patriotic groups).

5. *Rational Achievement (Orange)*: Seeks self-expression through their overt material accomplishments. Does not subjugate their opinions to a higher authority and often utilizes objective truths and scientific approaches as a vehicle for their accomplishments. Typically display little idealism and places personal success against the welfare of the group or the ecology.

Manifested in the following: capitalist entrepreneurs and corporate leaders.

6. *Sensitive Self (Green):* Seeks diverse and egalitarian nonhierarchical communities that acknowledge and value all perspectives above any single authority. Willing to subjugate their authority to others and has a strong sense of justice and attempts to reach consensus rather than subjugation. Concerned about ecological systems. Have high empathy skills and often values emotions above cognitive reasoning.

Manifested in the following: Postmodernism, nonprofits such as Greenpeace, animal rights groups, environmental activists, and human rights organizations.

### Second-Tier Value Lines

Clare Graves described second-tier values as indicating a quantum shift in human consciousness. Operating out of the second-tier level, the individual is able to recognize that each preceding level addresses some aspect of reality that is necessary to the development of human consciousness. In fact, any one of the first-tier levels may need to be activated in certain life conditions. Unlike each of the first-tier values that experience the world only through their blinkered perspective, the second tier includes and transcends the first-tier levels and do not experience a need to belong to any particular group. Rather than scarcity, individuals experience the universe and their own potential as abundant and limitless. Second tier signifies higher developmental stages of consciousness and is not to be confused with a particular state of consciousness.

7. *The Integrative Level (Yellow)*: This is characterized by flexibility, creativity, and spontaneity. Focuses on functionality rather than dogma and encourages the emergence of systems with increasing complexity.
8. *The Holistic Level (Turquoise)*: This is characterized by the motivation to support novel complex systems that support the emergence of compassionate and harmonious unification of the entire spectrum of human consciousness. This perspective is both idealistic and realistic and recognizes the specific needs of all previous levels.

Manifested in the following: Second-tier consciousness represents the leading edge of human consciousness and remains quite rare. Examples can be found in individuals such as Nelson Mandela, Gandhi, and Martin Luther King who midwifed profound cultural transformations.

It is very important to appreciate that the levels (stages) describe progressive and permanent landmarks along an evolutionary path that is manifesting the emergence of a more inclusive and complex unfolding of our potential. In this regard, integral theory offers a model for understanding the emergence of human flourishing. This is helpful to healers who should skillfully apply appropriate interventions suitable for a particular stage. For example, an individual who is experiencing values that are organized at a *mythic (blue)* level will be likely to accept interpretations and treatments that are framed in the context of receiving the blessing of a higher authority (eg, their appointed religious authority).

**Figure 2.**
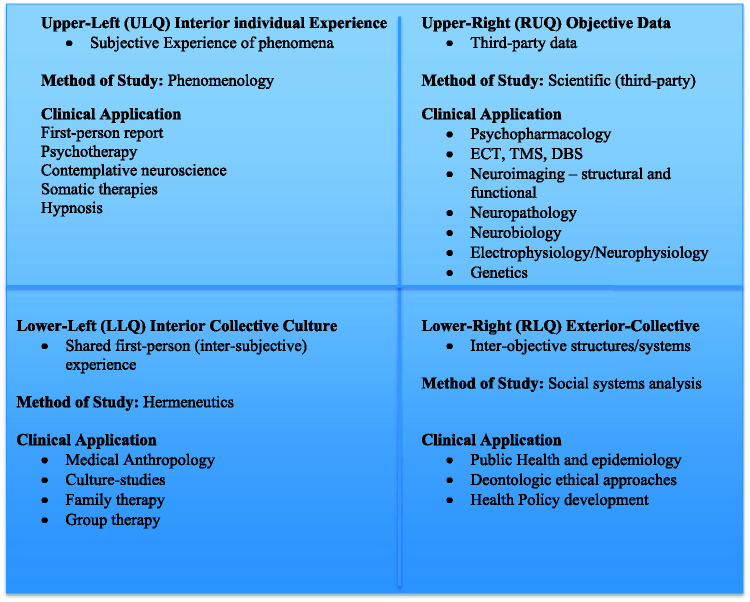
The Four Quadrants—Methodology and Clinical Applications in Mental Health.

Figure 2 describes the levels of development within each quadrant as proposed by Wilber.

## Lines (of Development)

The lines of development describe the capacities (“intelligences”) within each of the levels that manifest in each of the 4 quadrants. Each line has emerged in response to the challenges posed by life within different quadrants. Each person (and collective) demonstrates their own signature strengths and weakness in particular lines that can be plotted on a “psychograph.” Howard Gardner has developed the concept of “multiple intelligences” that include musical-rhythmic, visual-spatial, verbal-linguistic, logical mathematical, bodily-kinesthetic, interpersonal, intrapersonal, and naturalistic intelligences. An individual may demonstrate high intelligence in one line while also demonstrating significant weaknesses in another (eg, a sociopathic dictator who demonstrates high cognitive intelligence but low moral development). ^19^

Stated in more practical terms, the lines can be described as follows:

- Cognitive line: The complexity of one’s thinking.
- Moral line: The ability to discern how things should be.
- Emotional line: The capacity to experience and regulate emotions.
- Interpersonal line: The ability to relate to others in social situations.
- Self-identity line: The capacity to maintain a stable sense of personal identity.
- Aesthetic line: The capacity for experiencing and manifesting beauty.
- Spiritual line: The capacity to manifest one’s spiritual development.
- Values line: The capacity to experience increasingly prosocial values that shape one’s decisions. (The values line has been further developed into the SDI model—see later).

**Figure 3.**
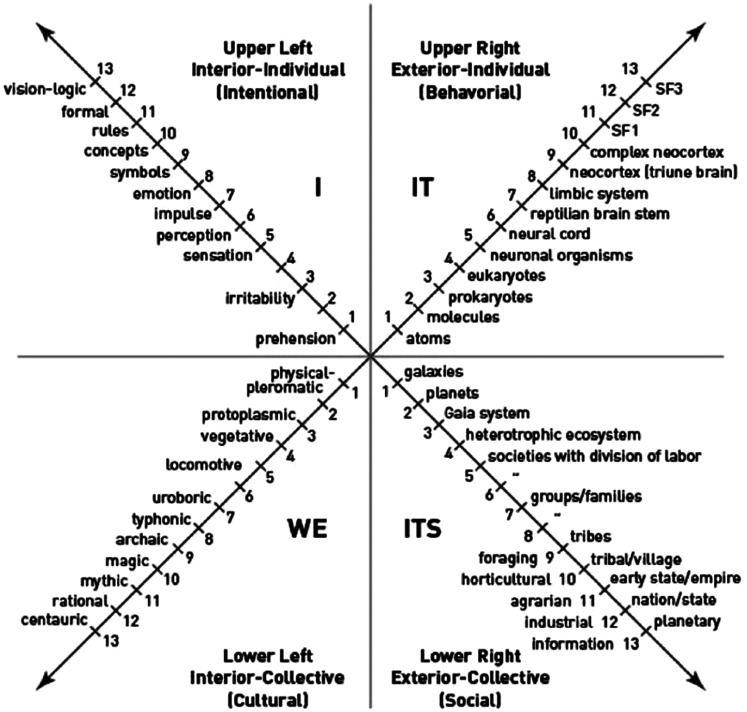
Levels within the four quadrants.^1^

**Figure 4.**
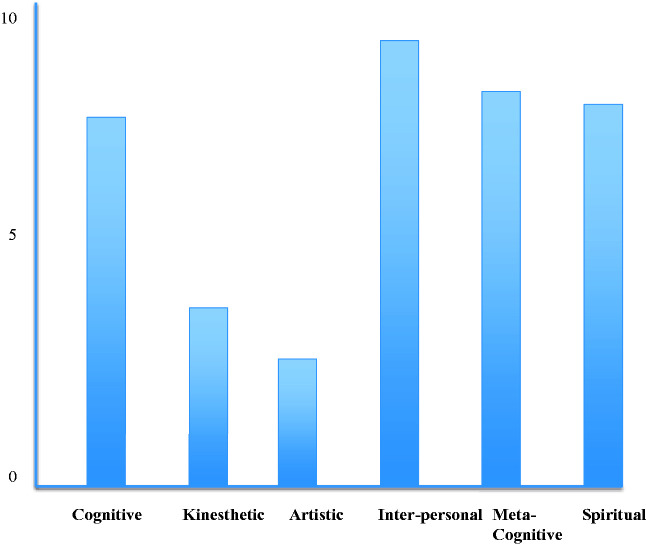
Example of a Psychograph.

Clinicians can assess their clients’ development across each line and develop a “psychograph” that enables them to identify and work skillfully with the individual’s (or groups) strengths and challenges (see Figure 3). Figure 4 is an example of a clinical “psychograph” that can be used to characterize an individual's strengths within different lines of development. For example, when working with an individual who has experienced significant developmental trauma it may be more effective to work within their somatosensory experience than focus on cognitive approaches.

## States (of Consciousness)

States are temporary states of consciousness such as waking, dreaming and sleeping, bodily sensations, and drug-induced and meditation-induced states. In contrast, structures are somewhat permanent patterns of consciousness and behavior. Levels and lines are representative of these structures of consciousness. The states describe vertical (spiritual) development, while the stages describe (psychological) horizontal development. Wilber has described this as “waking up” (state development) versus “growing up” (stage development).

The states manifested in each quadrant include the following:

LUQ States**:** These are states that are experienced from a first-person perspective includes as follows:

1. First-person feeling states (eg, such as elevated and depressed moods, insights, and intuitions).
2. The natural states of waking, dreaming, and deep sleep, and nondual states.
3. Meditative states induced by contemplative practices. These have been extensively explored by Eastern contemplative traditions. With sustained training they can move from being a state to a stable trait. Based on his thorough study of contemplative traditions, Wilber describes 4 types of consciousness, that is, gross, subtle, causal, and nondual.
4. Drug induces “altered” states.

RUQ States—These are states that can be observed by a third party:

5. Physical brain states (alpha, beta, theta, and delta waves) and hormonal states.
6. Behavioral states such as crying and smiling.
7. Physical states (eg, normal vs pathological, water versus ice)

LLQ States—These are consensus intersubjective states experienced by a group of individuals (mass hysteria, shared religious ecstasy, and the so-called “group think”) such as shared ecstasy and bliss or a communal experience of the divine.

LRQ States—These are states manifest by an ecological system. This notion of equilibrium is illustrative of various ecological states such as entropy (increased disorder) or eutrophy (being well-nourished).

## Types

Within the Integral model, the “Types” describe the stable patterns that manifest regardless of the developmental level of an individual or group. Examples of Types include one’s personality type, gender, or genotype. Since these Types are stable and resilient patterns, recognizing the characteristics of working within a specific Type is important when attempting to initiate sustainable change within an individual or collective. For example, rigidly attempting to employ monotheistic symbols within an atheistic culture will inevitably fail.

Here are some type characteristics that are distinguishing factors within the 4 domains as follows:

LUQ types: examples include personality and gender.

LLQ types: examples include different religious system (eg, monotheism, polytheism, and pantheism) and kinship systems (eg, Eskimo, Hawaiian, Lakota Sioux, and so on.).

RUQ types: examples include objectively measured types such as blood types, body types, and genotypes.

RLQ types: examples include types of governing (eg, democracy, dictatorship, oligarchies, and so on.)

## Practical Implications of an Integral Psychiatry Model for Mental Health Care

The Integral psychiatry model (IPM) provides a heuristic framework that has very practical clinical applications that can address the challenges facing the current mental health-care system. An integral clinical practitioner addresses these complex challenges by combining first-person, second-person, and third-person assessments, diagnostic formulation and methods, practices, and techniques in a given situation. Through this Integral approach, Integral practitioners are often able to identify vitally important insights into understanding and more effectively responding to the myriad factors that can influence the well-being of an individual or group.

The IPM addresses current deficiencies in contemporary psychiatry by the following:

1. *Expanding the paradigm beyond the current focus on the neurobiological domain*. As discussed earlier, the IPM recognizes the power and importance of third-person scientific discoveries (ie, “scientific truth”). However, it also recognizes the limitations of this monocular view and even its potential for harm when pursued in a vacuum that is blind to the experiential, social, and ecological determinants of human suffering. The IPM distinguishes between “curing” that occurs in the domain of scientific objectivity, and “healing” that manifests as enhanced coherence and development across and between each of the Integral elements. When necessary and appropriate, the IPM will certainly utilize biological treatments such as medications and interventional technologies. However, the IPM also values other therapeutic tools in the “Integral clinical toolbox” (eg, psychotherapies, contemplative practices, nutrition, environmental modifications, and social and regulatory changes) that offer the patient and their community a wider range of therapeutic options.
2. *Supporting an interdisciplinary team approach*. The Integral clinician should be proficient at identifying the multiple factors that influence their patient’s well-being. However, the integral clinician working within an IPM has the humility to recognize that they do not have the skills to effectively understand and address all of these factors. The Integral psychiatrist therefore works within a respectful nonhierarchical clinical team that possesses the skills and experience to develop and implement a treatment plan that will be most effective in addressing the patient’s suffering.
3. *Recognizing patterns, and not just the details*. The IPM assumes a wide perspective whose horizon is not blinkered by objective data points derived from psychometric tools and neurodiagnostic studies. The IPM does not disregard or minimize these objective data but places it in the broader context that supports insights into the relational patterns generated by the multiple interactions between psychological, experiential, and ecological factors that shape behavior. From this integral perspective, human behavior is recognized to be a manifestation of resonant patterns within ecological systems at both the micro and macro level.
4. *Promoting self-assessment, humility, and self-cultivation*. The Integral clinician is not held captive to a single dominating paradigm that characterizes a hierachical system. Rather, the Integral clinician recognizes the strengths and limitations of each paradigm in the context of their patient’s subjective experience and objective behavioral metrics. In this way, the IPM seeks to constantly assess and optimize a particular perspective and is motivated by compassion.
5. *Recognizing the importance of community engagement*. The IPM recognizes the importance of identifying the ecological factors that influence the well-being of their patients. This ecological perspective includes regulatory and legislative issues as well as the impact of the environmental factors (such as pollution). The Integral clinician will therefore recognize the vital importance of working beyond the walls of the clinic and engaging in positive community and legislative activism to improve the health of their community.
6. *Actively supporting human flourishing, rather than focusing on pathology*. Rather than simply attempting to “combat disease,” the Integral clinician is motivated by compassion to improve the flourishing of their patient. The IPM will therefore utilize positive psychology approaches that enhance optimism, gratitude, awe, and loving-kindness that enhance their patient’s inherent capacity for flourishing. Furthermore, the IPM attempts to identify and optimize each person’s particular strengths, rather than only focusing on their challenges. The IPM therefore empowers each individual to assume authority over their lives, rather than abdicating responsibility to medications or health-care delivery systems. IPM maintains an optimistic perspective at all times and recognizes that the evolutionary impulse is ultimately aligned toward wider and more coherent systems that support coherence and flourishing.
7. *Acknowledges and fosters individual and cultural diversity*. The IPM recognizes the first person and interpersonal frameworks shape the expression of neurobiology. With this insight, the IPM recognizes that diseases occur within the physical form of the body, but the meaning that persons and communities ascribe to this physical process becomes their subjective illness. A deep respect for the uniqueness of the individual and their culture are therefore central to the Integral clinician’s relationship to their work as healers. In this respect, the IPM fosters collaborative relationships with patients and their communities that are aligned with their patients’ values. This respectful collaboration is more likely to foster effective therapeutic approaches that will be accepted by that community.
8. *Recognizing the importance of first-person experience*. Rather than viewing first-person reports as qualitative data with limited scientific utility, the IPM actively seeks and respects the patient’s first-person experience as a vital information that should be incorporated into any diagnostic formulation and treatment plan. In this regard, the IPM recognizes that listening to and respecting the patient’s personal narrative is a crucial aspect of healing.
9. *Self-Cultivation*. The IPM recognizes the healer can only support healing to the level of their own personal development. Given this attitude, the integral clinician recognizes the importance of both self-cultivation as well as the acquisition of technical competence. The 4 pillars of an Integral healer are as follows: (1) Compassion, (2) Wisdom, (3) Competence, and (4) Self-cultivation.^20^ The Integral model provides a template for supporting a wisdom that transcends the limitations of dogma and prejudice. However, it also recognizes that this wisdom must be motivated by a compassionate intention that motivates the healer to acquire competence in their healing tradition. Given this attitude, the integral clinician recognizes the importance of both self-cultivation as well as the acquisition of technical competence. Through self-cultivation techniques such as contemplative practices, physical self-care, nutrition, and environmental supports, the Integral healer enhances their resilience and reduces the likelihood of experiencing burnout.

## Limitations of Integral Theory

Although Integral theory has found acceptance across many arenas (including business, education, and politics), it has garnered detractors who criticize it for simply being a model and not describing a practical method for supporting positive evolutionary change. This argument in itself is not a criticism of Integral theory but does highlight the challenges inherent to supporting change within any system. The SDI model has however addressed this challenge by providing practical approaches for understanding how to understand facilitate change.^18^

The Integral theory model has also been criticized for failing to recognize that biological systems are not static but exhibit evolutionary changes that will influence the so-called “scientific truth” of the neuroscientific method. This is illustrated by the finding that sustained contemplative practices produce measurable structural and connectivity changes in meta-relational structures such as the insular cortex.^21^ These changes will in turn influence the individual’s response to changes in the internal and external environment.

## Concluding Remarks

The IPM provides an elegant and comprehensive map for understanding and responding to the enormous challenges facing contemporary psychiatry in the 21st century. Utilizing this model, integral mental health care values the importance of the biomedical model but also recognizes that it is not sufficient to understanding and supporting human flourishing. It also provides a template for the development of a more effective and compassionate integrative approach to mental health care that is vital to navigating the challenges manifesting in the early 21st century. However, although it strives to describe the myriad factors that shape behavior, the IPM appreciates that its ultimate goal is to support the manifestation of coherent wholeness that supports human flourishing. Wiliam James captures this perspective when he wrote,

The **oneness** of things, superior to their **manyness**, you think must also be more deeply true, must be the more real aspect of the world. . . . The real universe must form an unconditional unit of being, something consolidated, with its parts co-implicated through and through.^22^—William James, 1907
